# Face-to-face versus distance learning of basic suturing skills in novice learners: a quantitative prospective randomized trial

**DOI:** 10.1186/s12909-022-03353-3

**Published:** 2022-04-18

**Authors:** Ahmad Zaghal, Charles Marley, Salim Rahhal, Joelle Hassanieh, Rami Saadeh, Arwa El-Rifai, Taha Qaraqe, Martine ElBejjani, Rola Jaafar, Jamal J. Hoballah

**Affiliations:** 1 Department of Surgery, Division of General Surgery, American University of Beirut-Medical Center, Beirut, Lebanon; 2 Department of Clinical Psychology, University of Edinburgh, Edinburgh, UK; 3 Clinical Research Unit and Department of Internal Medicine, American University of Beirut-Medical Center, Beirut, Lebanon

**Keywords:** Distance learning, Face-to-face learning, Medical education, Suturing skills, Pandemic

## Abstract

**Background and aim:**

Traditionally, practical skills are taught on face-to-face (F-F) basis. COVID-19 pandemic brought distance learning (DL) to the spotlight because of the social distancing mandates. We sought to determine the acceptability and effectiveness of DL of basic suturing in novice learners.

**Methods:**

A prospective randomized controlled trial involving 118 students was conducted. Participants were randomized into two groups for learning simple interrupted suturing: F-F and DL-groups. Evaluation was conducted by two assessors using a performance checklist and a global rating tool. Agreement between the assessors was calculated, and performance scores of the participants were compared. Participants’ satisfaction was assessed via a questionnaire.

**Results:**

Fifty-nine students were randomized to the F-F group and 59 to the DL-group. Satisfactory agreement between the assessors was demonstrated. All participants were successful in placing three interrupted sutures, with no significant difference in the performance between the groups. 25(44.6%) of the respondents in the DL-group provided negative comments related to the difficulties of remotely learning visuospatial concepts, 16(28.5%) preferred the F-F approach.

**Conclusion:**

DL of basic suturing is as effective as the F-F approach in novice learners. It is acceptable by the students despite the challenges related to the remote learning of practical skills.

## Introduction

### COVID-19 pandemic has accelerated the adoption of distance education

Over the past two decades, there has been a global tendency to maximize the utilization of distance learning; this trend is supported by the recent advances in the web-based technologies, and precipitated by the need to provide reliable, equitable, efficient, and cost-effective education [40]. Despite its promising characteristics, many educational institutions were slow at adopting distance education until the COVID-19 era erupted, which prompted many medical schools to pull their students from clinical clerkships to protect them from acquiring the infection and preserve the scarce supplies of personal protective equipment [14] resulting in an abrupt critical reduction in the students’ clinical exposure. This unprecedented event prompted many educational programs to transition to remote learning [35] with an exponential rise in the innovative utilization of information technology and web-based instructional methods in education [61]. This was mirrored in medical education, with the majority of medical programs resorting to web-based remote teaching strategies to maintain the integrity of their medical students’ education [22].

### Medical educational concerns during the pandemic

Because of the lack of experience in online teaching of medical knowledge and skills, and scarcity of the data available on its effectiveness, medical educators and medical students have voiced concerns about the impact of this abrupt transition to distance learning on the quality of medical education and the future of healthcare [8, 58]. The clinical component of medical education has always relied heavily on hands-on experience such as history taking and performing physical exam, hence the heightened concerns that remote learning of these skills may not be sufficient for the acquisition of optimal competency and confidence for medical students [14].

### Remote teaching of basic surgical skills

Acquiring basic surgical skills is recognized as an important element of undergraduate curricula in many medical schools worldwide [38]; this has been highlighted by the graduate medical council of the UK [28]. Teaching technical skills is a particularly difficult component of surgical education to achieve remotely [54] owing to its heavy reliance on physical interactions and immediate technical feedback between the teachers and learners. Traditionally, learning basic surgical skills such as simple interrupted suturing occurs in a simulated environment utilizing part-task trainers to allow the learners to achieve competency in suturing by deliberate practice under direct face-to-face tutoring and supervision. This physical interaction became impossible during the extended periods of lockdown imposed by the COVID-19 pandemic, which prompted many educators to utilize innovative tools such as teleconferencing and take-home simulation kits to facilitate remote learning of practical skills to make up for the lost learning opportunities during the lockdown [34]. Since then, many authors have reported on their experience with distance learning of surgical skills; a recent prospective case-control study [19] concluded that the outcomes of web-based learning of basic suturing were comparable to the traditional face-to-face approach in a cohort of 62 medical students.

At our hospital, during the initial lockdown period in March and April 2020, all medical students’ clinical clerkships were temporarily cancelled, and their educational activities switched to web-based remote learning, utilizing teleconferencing and commercially available web-based interactive clinical modules. Teaching surgical technical skills, such as basic suturing, presented itself as a more challenging component of the clinical education under these unusual circumstances as compared to teaching cognitive skills and knowledge, which raised questions and concerns around the effectiveness and acceptability of distance learning of basic surgical skills. There exists no clear guidance in the literature on how to best design and deliver basic surgical skills teaching sessions remotely to novice learners; this is because the world has not witnessed a situation similar to the COVID-19 pandemic in modern history. Hence, like many other educators, we had to improvise and use the available technology and expertise to deliver the basic suturing skills sessions to our students. We utilized WebEx teleconferencing platform to deliver synchronous online teaching sessions whereby the tutors demonstrate the suturing skills, in a simulated setting, to the students who in turn practice suturing (remotely) and receive real-time feedback on their performance. This was our first experiment with online instructional methods for delivering practical skills sessions. The post-course surveys revealed that although the students enjoyed and benefited from the sessions, the tutors felt that the online approach was more demanding than its conventional “face-to-face” counterpart, particularly when it came to explaining certain concepts that relied on visuospatial abilities, such as teaching the proper technique of mounting the needle on the needle holder.

### Aims and research question

In this study, we primarily sought to compare the acceptability and effectiveness of distance learning of basic suturing to the classic face-to-face approach in novice learners, which is currently considered the standard approach of teaching surgical skills to medical students. The specific research questions the study aims to answer is as follows: Is distance learning acceptable and effective in learning simple interrupted suturing in novice learners as compared to the traditional face-to-face approach?

## Research design and methods

This is a prospective randomized controlled trial involving two arms (face-to-face and distance groups) of learning simple interrupted suturing in pre-medical and first and second-year medical students in a simulated setting using a part-task trainer. Immediately after the teaching sessions, two independent surgeons assessed the students’ simple interrupted suturing performances using a validated checklist and a validated OSATS global rating tool. We compared the participants’ performances in each arm to determine the effectiveness of distance learning of basic suturing as compared to the traditional face-to-face instructional method. In addition, the students in both groups were asked to complete a questionnaire, immediately after the teaching sessions, to evaluate their satisfaction (acceptability) and confidence. The study ended once the complete set of participants has been recruited.

### Participants

Pre-medical, first, and second-year medical students from a four-year Doctor of Medicine graduate-entry programme. Premedical students include senior biology, medical laboratory, and nutrition students. All participants had no previous experience with suturing. Basic suturing is one of the educational activities of the third-year medical students, before which students are not normally exposed to basic suturing skills teaching sessions. The participants were recruited via an email sent to all the potential candidates for participation in this study.

#### Inclusion criteria

Pre-medical, first, and second-year medical students with no previous experience in suturing.

#### Exclusion criteria

Previous experience in suturing.

#### Randomization process

Computer-generated randomization was performed.

### Instructional methods

A right-handed surgeon with 12-year experience in teaching surgical skills ran all the teaching sessions in both groups. The sessions, for both groups, were designed based on Kneebone [37] recommendations for teaching technical surgical skills.

### ***Control group:*** face-to-face learning of simple interrupted suturing

- The students watched a video demonstrating simple interrupted suturing, with the instructor commenting on the steps.
- The students then watched the video again.
- The instructor then demonstrated the procedure for the students.
- The students then practiced suturing with immediate and specific feedback provided by the instructor until he and the students were satisfied with the performance.

#### ***Study group:*** distance learning (tele simulation) of simple interrupted suturing

- The instructor ran the interactive tele simulation sessions utilizing web-based video-conferencing technology (WebEx platform). The students used their personal smartphones or laptops with audio-video capabilities. The instructor ran the session through his smartphone.
- The instructor shared a video demonstrating simple interrupted suturing while commenting on the steps (the same video used in the control group).
- The instructor then ran the video again for the students.
- The instructor then demonstrated the skill for the students by turning on his camera.
- The students then practiced suturing, and periodically turned on their cameras to receive live and specific feedback from the instructor on their performance, until the instructor and the students were satisfied.
- No face-to-face interactions between the students and the instructor.

To avoid poor internet connectivity issues, the intervention group sessions were run on hospital premises to use the reliable institutional internet connection; the participants used personal headsets and their own computers/smartphones and were seated at least three meters apart to maintain privacy and social distancing.

**Fig. 1:**
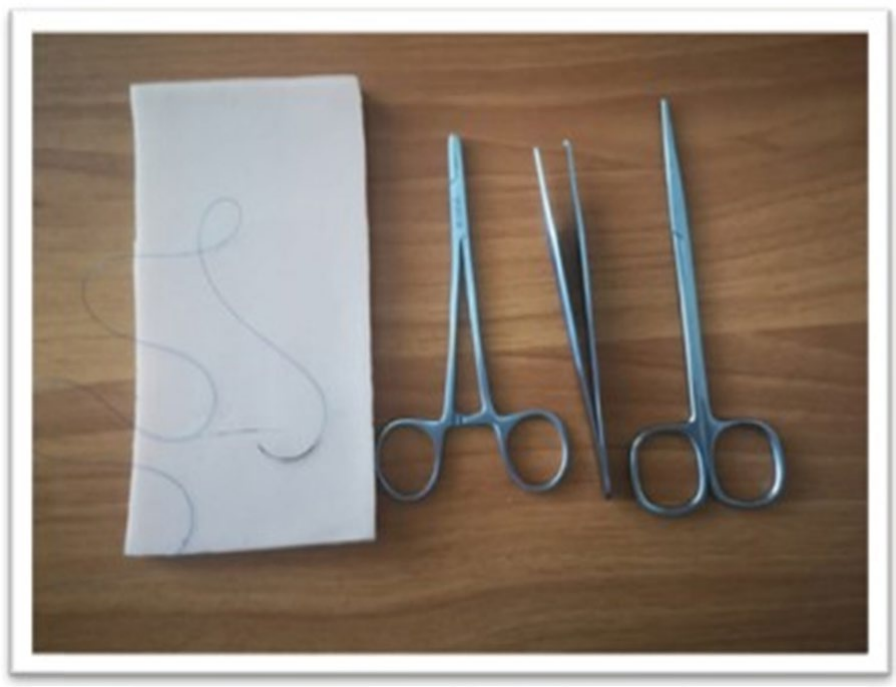
Silicon suturing pad and surgical instruments used in both groups

The following material were provided to the students during the sessions in both groups (Fig. 1):

- A silicon suturing pad
- 3–0 Nylon suture
- One needle driver
- One pair of toothed forceps
- One pair of suture scissors
- A sharp dispenser

A publicly available YouTube video was utilized [66] after obtaining the approval of its author via email communication; the video’s content has been previously reviewed by our group and has routinely been used during practical suturing sessions for the third-year medical students. The participants in both groups were not given the link of the instructional video prior to attending the sessions. The same video was used in both groups.

### Data collection

The participants’ demographic data was collected via a brief questionnaire that asked about age, gender, hand-dominance, and whether they play musical instruments.

#### Primary outcome measures

**Fig. 2:**
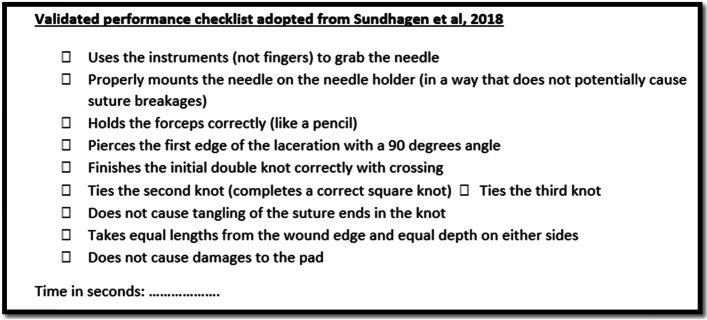
Performance checklist: adopted from [68]

**Fig. 3:**
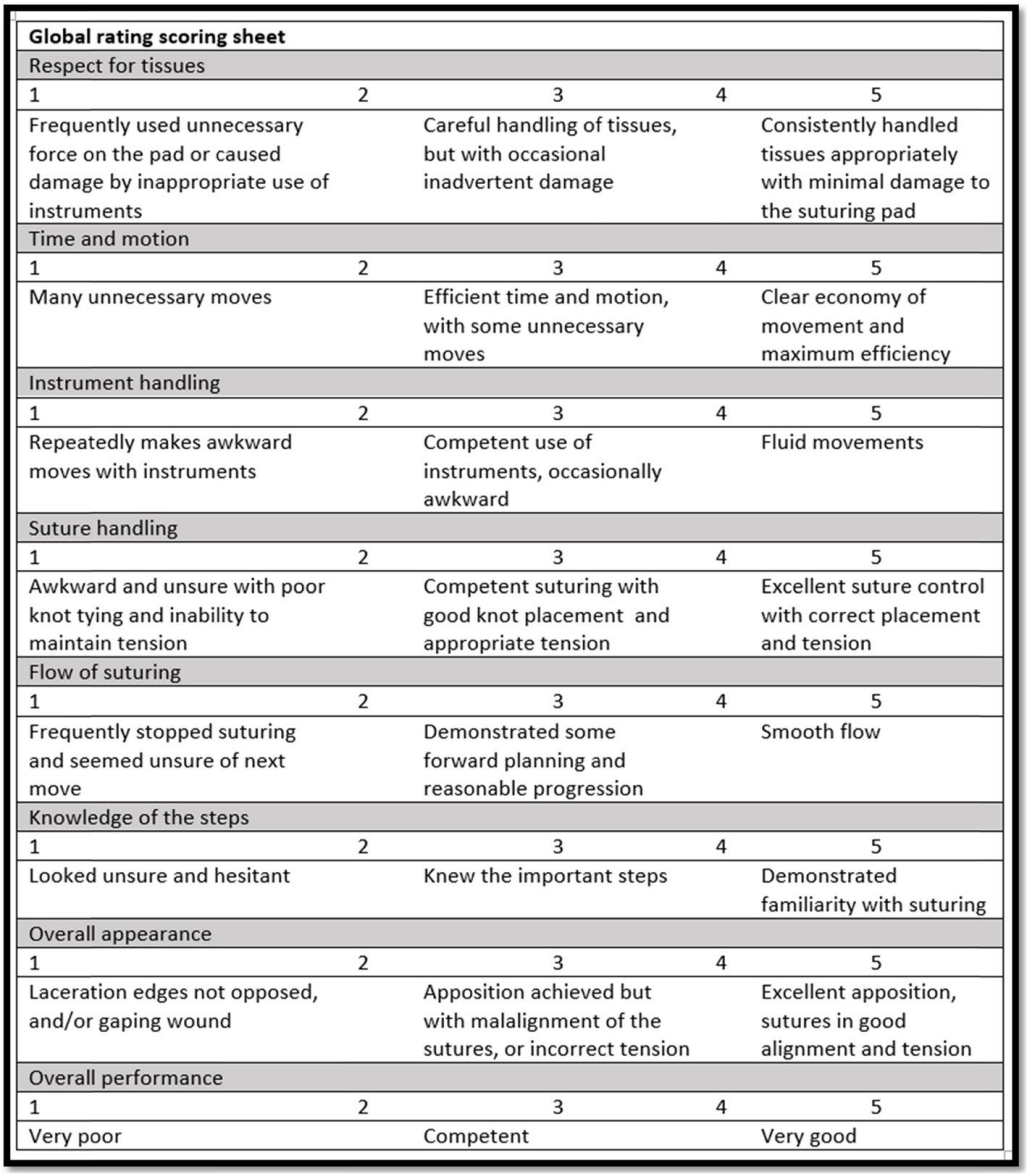
Global rating scoring sheet (OSAT): adopted from [1]

Immediately after the sessions, students in both groups were asked to place and tie three simple interrupted sutures using the same suturing pads and surgical instruments they used during the sessions. The participants were provided with clearly written instructions explaining the assessment process. Assessment time was limited to 10 min based on our previous experience with OSCE suturing stations for students. The research assistant individually videotaped the participants, using a high-resolution smartphone camera, while performing the suturing tasks without any guidance or interference. The instructor and the assessors did not participate in the video-recording process. The video frame included only the suturing pads and the participants’ gloved hands. All video recordings were muted and deidentified before being sent to the assessors for grading using a validated performance checklist [68] (Fig. 2) and a validated Objective Structured Assessment of Technical Skills (OSATS) global rating sheet (Fig. 3). Time lag between the sessions and assessment was controlled, whereby all the assessment-recordings were performed within five to twenty-five minutes after the conclusion of each session.

##### Assessors

Two right-handed independent surgeons with extensive experience in teaching basic suturing skills independently evaluated the video-recordings of the students’ performances, each utilizing the two assessment tools mentioned above.

##### Blinding process

To minimize bias, namely observer bias [31], the assessors were blinded to the participants’ identities and instructional groups [63]. The instructor did not participate in the video recording and assessment of the students’ performances.

##### Deidentification process

All video recordings were deidentified by assigning random numbers to them. The research assistant safeguarded the deidentification cipher; neither the instructor nor the assessors had access to the deidentification cipher.

##### Primary outcome measurement tools

- We used a validated ten-item checklist for assessing suturing skills in medical students developed by Sundhagen et al. [68] (Fig. 2); the authors proved that their checklist can satisfactorily differentiate between novice and expert performances (construct validity) and showed a narrow variation in the scores provided by three independent expert assessors (inter-rater reliability). Overall performance scores were calculated based on the formula used by the same authors: “**cutoff time (seconds) – completion time (seconds) – (10 x sum of errors)”** ([68]; p 209) with higher scores indicating better performance; this formula was also previously used and validated by other authors [29].
- OSATS is a validated observational assessment tool of surgical skills ([45]; Faulkner et al. 1997 [1];) particularly in postgraduate trainees; it has also been used in medical students [62]. It comprises of seven performance domains whereby learners are scored on a 5-point Likert scale on proper tissue handling, efficiency and economy of movements, instrument handling, suture handling, flow of the procedure, knowledge of the steps of the procedure, overall appearance of the suture, in addition to an overall assessment of the performance; higher scores indicate better performance. Figure 3 depicts the OSATS global rating score sheet adopted from Alam et al. [1] that was used in this study. If designed well, OSATS can carry out its intended task of measuring the levels of the participants’ performances (valid) [32], and can generate comparable scores when repeated by different examiners (reliable) [45].

#### Secondary outcome measurement tool

**Fig. 4:**
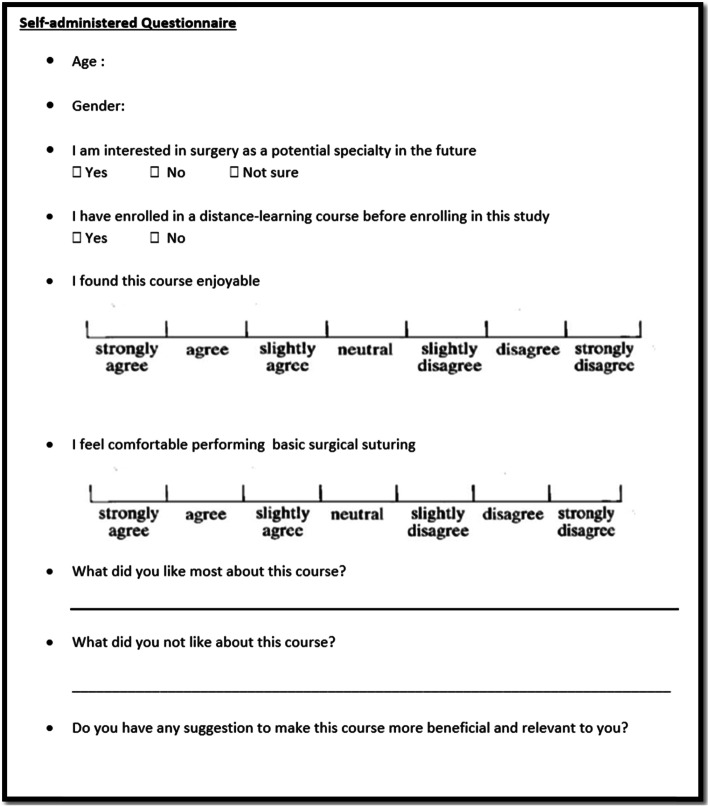
Self-administered questionnaire

The students were asked, on voluntary basis, to electronically fill a brief anonymous questionnaire after the completion of their respective sessions to evaluate their satisfaction (acceptability), confidence levels, and attitudes towards the instructional method they experienced (Fig. 4). We followed the AMEE’s guide No.87 [5] recommendations for the survey items development, making sure to use clear self-explanatory language, avoid using negative-worded sentences, and include verbal labels for all the Likert-scale responses of the closed-ended items. We have also included three open-ended questions to gain a more detailed grasp of the students’ perceptions of their learning experience. No Any changes to trial outcomes were made after the trial commenced.

### Sample size

We have utilized a free online statistical calculator to determine the minimum sample size required to provide adequate power in comparing the participants’ scores between the two arms of the study [16]. At least 116 participants are required to ensure a 90% power and a type-I error (Alpha) of 0.05. This was based on an anticipated 10% difference in the participants’ checklist formula scores in favour of the F-F approach. The estimation of this percentage difference was made arbitrarily because of the scarcity of published data on this matter, with most of the available evidence point to a small difference between face-to-face and remote learning of practical skills. All first and second-year medical students as well as pre-medical students serving at the hospital (a pool of 439 students) were invited to participate in the study. We enrolled the first 118 students who volunteered to participate in the study.

### Ethical considerations and confidentiality issues

We have obtained ethical approval from the local institutional review board before starting the recruitment process. All pre-medical, first, and second-year medical students were invited to participate in the study; all students were given equal opportunities to contribute to the study.

### Statistical analysis

Statistical analysis was carried out through statistical package for the social sciences (SPSS) software. Data was exported from Microsoft excel (Excel V.16.29, 2019) to SPSS. Statistical tests are stated in the same order of the reported results. Categorical variables were presented as frequencies and percentages [N (%)], and continuous data as mean ± standard deviation (Mean ± S.D).

We first compared the scores between the two assessors to check for inter-rater variability, by using the Chi square test for the categorical variables (checklist and OSATS items) and Student t-test for the continuous variables (checklist formula scores and OSATS total scores) between the two assessors. For each participant in each arm, we used the average rating per each graded checklist item, checklist formula score, and OSATS total score to carry the subsequent analysis on the participants’ performances. Chi-square tests were then used to assess whether the graded checklist items were different between the two study arms (face-to-face versus distance learning). Independent Student t-test was used to compare the two groups for continuous dependent variables including the mean scores (checklist formula score, and OSATS total score). Results were considered statistically significant at *p* < 0.05.

Regarding the time needed by the students to complete the assessment task, the range between the lowest and the highest reported time was wide, and the distribution of the data was moderately skewed, with a skewness value of 0.668 [12]. Hence, the median was used instead of the mean. Mann-Whitney U test was used to compare between the two groups. Results were considered statistically significant at *p* < 0.05.

The participants’ responses to the questionnaire were presented in bar charts and described. Some students offered narrative comments in the questionnaire; those were also described. A formal thematic analysis was not performed because of the relative simplicity and small number of the comments.

## Results

### Demographic data

Between January and May 2021, 118 medical and pre-medical students with no prior experience in basic suturing consented to the study. Fourteen F-F and seventeen DL sessions were conducted; each session engaged a small group of 2 to 8 students based on their availability. Two to 8 students (mean: 4.1) in the F-F group and 2 to 7 students (mean: 3.4) per session in the DL group.

Table 1 depicts the demographic data of the participants. There were no statistically significant differences in the demographic parameters between the two groups. The participants’ mean age was 21.47 [18–27] years and 21.42 [18–26] years in the F-F and DL groups respectively (*p* = 0.53). 32(53.3%) of the participants were females in the F-F group versus 28(46.7%) in the DL group (*p* = 0.46). Similarly, there were no statistical differences in the seniority level, hand-dominance, and experience in playing musical instruments between the two groups.

**Table 1:** Demographic data

Demographic data	Total number of participants (***n*** = 118)		***p***-value
	Face-to-face learning (***n*** = 59)	Distance learning (***n*** = 59)	
**Mean age (range) in years**	21.47 (18–27)	21.42 (18–26)	**0.55**
**Gender**			
Female	32 (53.3%)	28 (46.7%)	**0.46**
Male	27 (46.6%)	31 (53.4%)	
**Seniority level**			
Senior Pre-medical students	17 (28.8%)	17 (28.8%)	**0.97**
First-year medical students	21 (35.6%)	20 (33.9%)	
Second-year medical students	21 (35.6%)	22 (37.3%)	
**Hand-dominance**			
Right	57 (96.6%)	52 (88.1%)	**0.08**
Left	2 (3.4%)	7 (11.9%)	
**Plays a musical instrument**	16 (27.1%)	16 (27.1%)	**0.49**

### Inter-rater reliability

Tables 2 and 3 present the percentages of the students who successfully demonstrated the checklist and OSATS performance items and the average overall scores by both assessors. For the checklist items (Table 2), the assessors’ evaluations were highly concordant (with all *p*-values from chi-square tests for the checklist items > 0.05); the checklist formula scores were also highly similar (*p* = 0.96 from student t-test) between the two assessors. Similarly, for the OSATS scores (Table 3), there were no prominent differences between the assessors with *p*-values > 0.05 for all the sheet items except two (time and motion, and overall appearance), with no statistically significant difference in the total scores between the two assessors (*p* = 0.27).

**Table 2:** Inter-rater reliability: checklist

	Assessor 1	Assessor 2	***p***-value
**Uses instruments correctly** ^a^	91 (77.1%)	92 (78%)	**0.84**
**Mounts needle correctly** ^a^	97 (82.2%)	95 (80.5%)	**0.69**
**Holds forceps correctly** ^a^	100 (84.7%)	97 (82.2%)	**0.44**
**Pierces at 90°** ^a^	112 (94.9%)	109 (92.4%)	**0.40**
**Double knot-crossing** ^a^	116 (98.3%)	116 (98.3%)	**1**
**Second knot** ^a^	116 (98.3%)	115 (97.5%)	**0.65**
**Third knot** ^a^	116 (98.3%)	117 (99.2%)	**0.31**
**No tangles** ^a^	102 (86.4%)	100 (84.7%)	**0.61**
**Equal wound edges** ^a^	104 (88.1%)	98 (83.1%)	**0.18**
**No damage to pad** ^a^	101 (85.6%)	105 (89.0%)	**0.39**
**Checklist formula score (Mean ± S.D)**	317.18 ± 62.99	317.41 ± 64.57	**0.96**

^a^N (%) of participants who correctly demonstrated the checklist item

**Table 3:** Inter-rater reliability: OSATS global rating

OSATS global rating	Assessor 1	Assessor 2	***p***-value
**Respects tissues** ^a^			
1	0 (0%)	3 (2.5%)	**0.06**
2	9 (7.6%)	8 (6.8%)	
3	60 (50.8%)	47 (39.8%)	
4	45 (38.1%)	48 (40.7%)	
5	4 (3.4%)	12 (10.2%)	
**Time and motion** ^a^			
1	1 (0.8%)	2 (1.7%)	0.02*
2	9 (7.6%)	7 (5.9%)	
3	65 (55.1%)	57 (48.3%)	
4	39 (33.1%)	39 (33.1%)	
5	4 (3.4%)	13 (11.0%)	
**Instrument handling** ^a^			
1	0 (0%)	4 (3.4%)	**0.21**
2	19 (16.1%)	22 (18.6%)	
3	51 (43.2%)	43 (36.4%)	
4	38 (32.2%)	39 (33.1%)	
5	10 (8.5%)	10 (8.5%)	
**Suture handling** ^a^			
1	0 (0%)	1 (0.8%)	**0.16**
2	23 (19.5%)	14 (11.9%)	
3	47 (39.8%)	53 (44.9%)	
4	37 (31.4%)	36 (30.5%)	
5	11 (9.3%)	14 (11.9%)	
**Flow of operation** ^a^			
1	0 (0%)	1 (0.8%)	**0.67**
2	5 (4.2%)	14 (11.9%)	
3	59 (50.0%)	53 (44.9%)	
4	41 (34.7%)	36 (30.5%)	
5	13 (11.0%)	14 (11.9%)	
**Knowledge of procedure** ^a^			
1	1 (0.8%)	3 (2.5%)	**0.76**
2	5 (4.2%)	7 (5.9%)	
3	44 (37.3%)	38 (32.2%)	
4	46 (39.0%)	42 (35.6%)	
5	22 (18.6%)	28 (23.7%)	
**Overall appearance** ^a^			
1	0 (0%)	0 (0%)	0.00*
2	6 (5.1%)	4 (3.4%)	
3	56 (47.5%)	41 (34.7%)	
4	37 (31.4%)	45 (38.1%)	
5	19 (16.1%)	28 (23.7%)	
**Overall performance** ^a^			
1	0 (0%)	2 (1.7%)	**0.89**
2	7 (5.9%)	7 (5.9%)	
3	52 (44.1%)	48 (40.7%)	
4	36 (30.5%)	35 (29.7%)	
5	23 (19.5%)	26 (22.0%)	
**OSATS total score (Mean ± S.D)**			
27.47 ± 5.85		28.05 ± 6.69	**0.27**

*: statistically significant (*p*-value< 0.05)

^a^N (%) of participants who correctly demonstrated the OSATS item

### Duration of the sessions

The mean duration of the F-F sessions was 80.93 ± 17.086 min versus 89.39 ± 30.287 min in the DL group, the difference was not statistically significant (*p* = 0.17).

### Time to complete the assessment task

The participants required a median of 282 s (range: 138–504 s) to complete the assessment task in the F-F group versus 248 s (range: 159–438 s) in the DL group. This was statistically significant (U = 5596, *p* = 0.01).

### Performance scores

None of the participants in both groups failed the exam; they all were able to place three secure interrupted sutures in less than 10 min.

Table 4 illustrates the differences in the students’ performances in the F-F versus DL groups, whereby the distribution of the students who succeeded in correctly demonstrating the checklist-items was compared between the two groups. There were no statistically significant differences between the two groups across all the items of the checklist.

**Table 4:** Participants’ performances in the face-to-face versus distance learning group: checklist items

Checklist item	Face-to-face (***N*** = 59)	Distance learning (***N*** = 59)	***p***-value
**Uses Instruments correctly** ^a^	45 (76.3%)	44 (74.6%)	**0.83**
**Mounts needle correctly** ^a^	48 (81.4%)	43 (72.9%)	**0.27**
**Holds forceps correctly** ^a^	48 (81.4%)	46 (78)	**0.64**
**Pierces at 90°** ^a^	55 (93.2%)	54 (91.5%)	**0.72**
**Double knot-crossing** ^a^	59 (100%)	59 (100%)	**1**
**Second knot** ^a^	57 (96.6%)	57 (96.6%)	**1**
**Third Knot** ^a^	57 (96.6%)	58 (98.3%)	**0.55**
**No tangles** ^a^	49 (83.1%)	48 (81.4%)	**0.81**
**Equal wound edges** ^a^	50 (84.7%)	48 (81.4%)	**0.62**
**No damage to the pad** ^a^	48 (81.4%)	51 (86.4%)	**0.45**

^a^N (%) of participants who correctly demonstrated the checklist item

Table 5 depicts the differences in the overall performance scores between the two groups. The participants in the F-F group slightly outperformed their peers in the DL group based on the checklist formula scores (326.10 ± 42.54 versus 306.05 ± 65.03); however, this did not reach statistical significance (*p* = 0.06). Similarly, there was no significant difference in the OSATS total scores between the two groups: 27.54 ± 5.99 versus 27.98 ± 5.24 (*p* = 0.63).

**Table 5:** Participants’ performances in the face-to-face versus distance learning group: checklist formula score and OSATS total score

	Face-to-face learning	Distance learning	***p***-value
**Checklist formula score** (Mean ± S.D)	326.10 ± 42.54	306.05 ± 65.03	**0.06**
**OSATS total score** (Mean ± S.D)	27.54 ± 5.99	27.98 ± 5.24	**0.63**

### Participants’ satisfaction and confidence

**Fig. 5:**
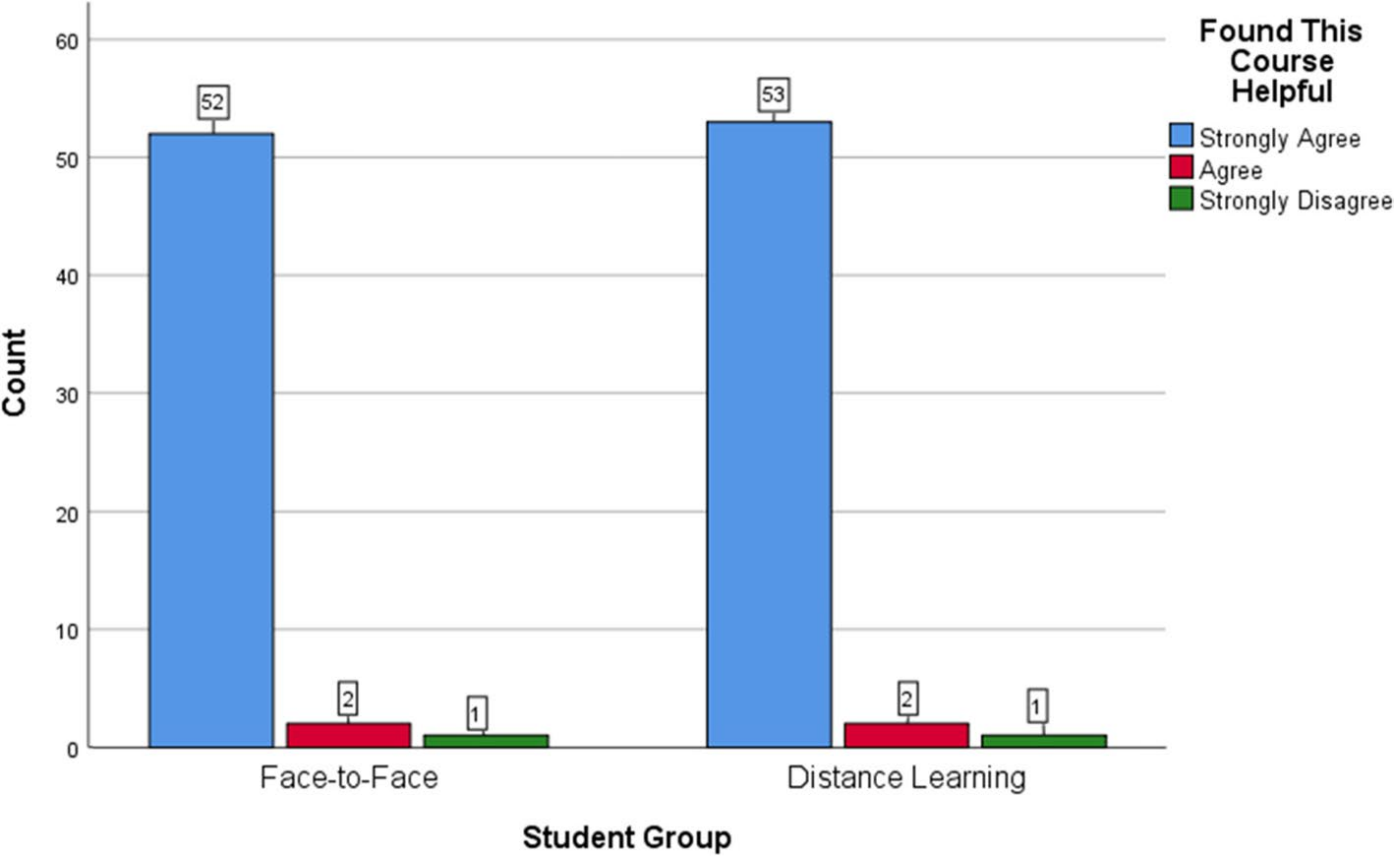
Face-to-face versus distance learning: participants’ perspectives on the usefulness of the sessions

**Fig. 6:**
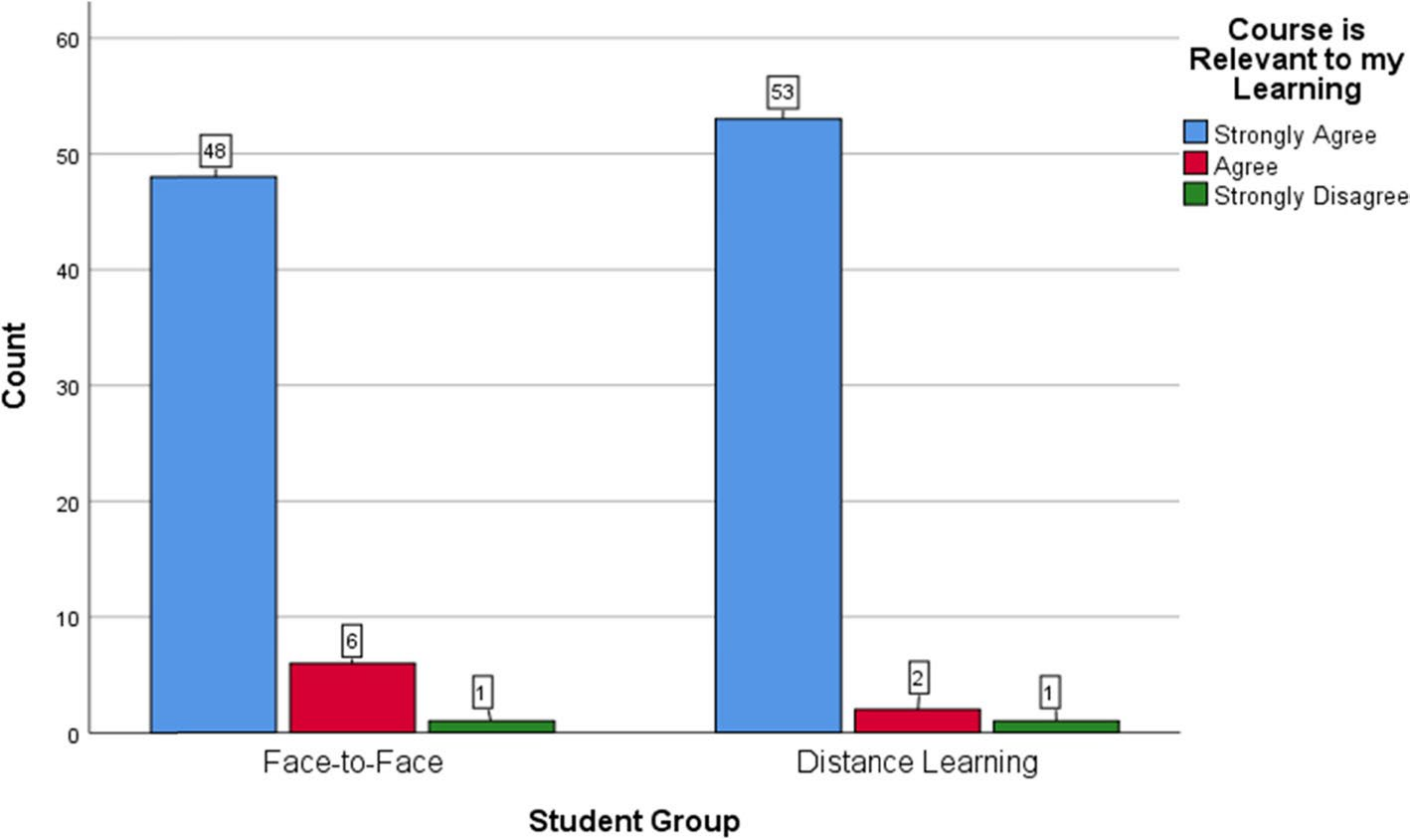
Face-to-face versus distance learning: participants’ perspectives on the relevance of the sessions

**Fig. 7:**
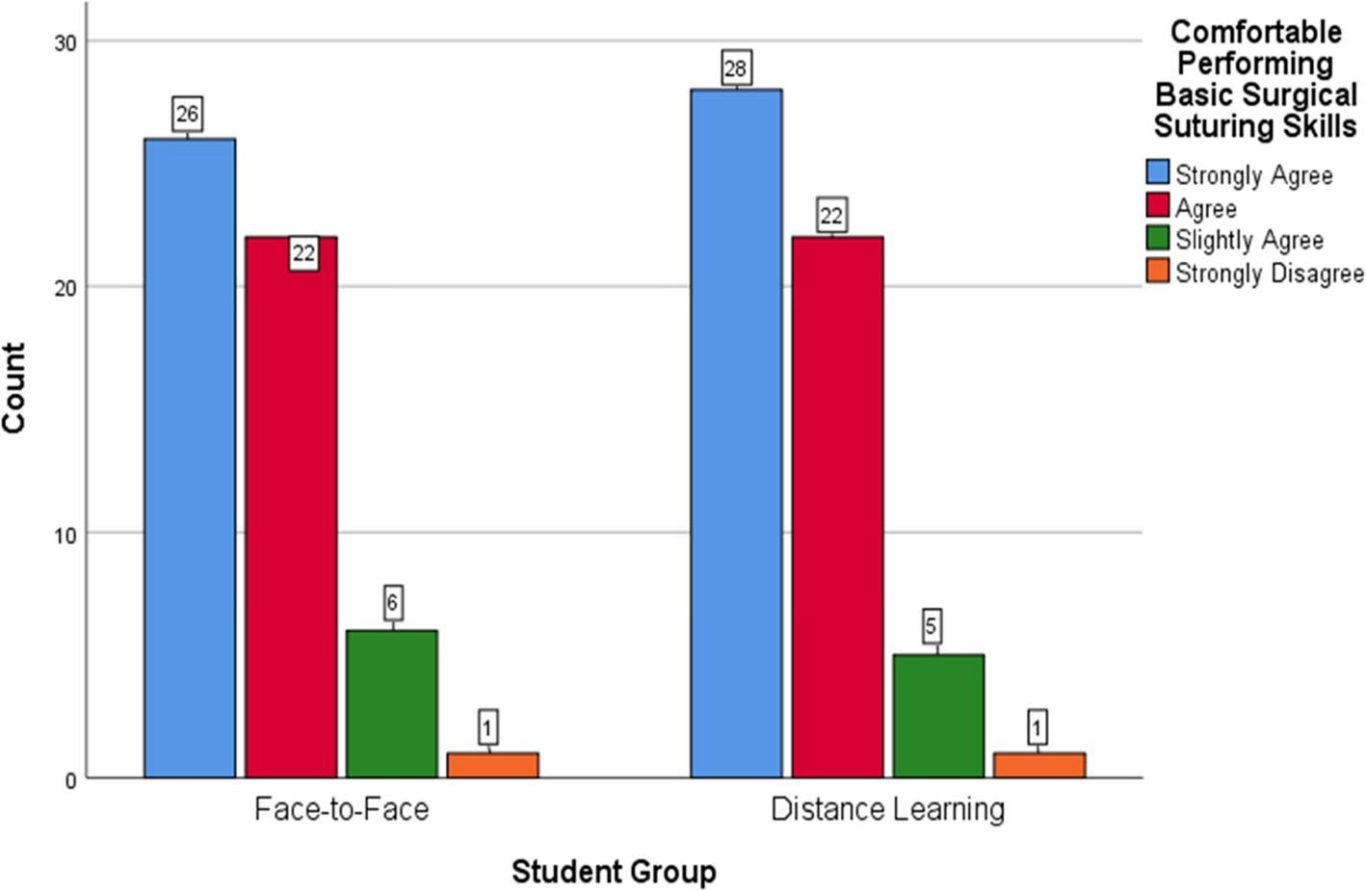
Face-to-face versus distance learning: participants’ confidence

One hundred and elven (94%) participants completed the questionnaires at the end of the teaching episodes. Overall, most of the students in both groups positively rated the sessions, found them helpful and relevant to their learning, and felt confident in performing basic suturing. The trends of positive responses were similar in the two groups. Figures 5-7 offer a visual demonstration of the comparison of the students’ responses to the questionnaires between the two groups.

Most of the students provided narrative comments by answering the open-ended questions of the surveys. Almost all the comments in the F-F group were positive and related to the effectiveness of the sessions, usefulness of the feedback, and how much they enjoyed the sessions: *“It is a great experience for future doctors”, “Learned a lot! Very helpful and informative session of important skills to learn”*. Similar positive comments came from almost all the students in the DL group: *“Session was interactive, and instructions were straightforward and easy to grasp”, “It was very informative and enjoyable”.* However, 25/56 students (44.6%) in the DL group provided negative comments that mainly revolved around internet connectivity and the visuospatial difficulties of learning surgical skills without the physical presence of the proctor: “*Bad Connection/No electricity ports”,* “*It was a bit difficult to understand how to hold the instrument*”, *“The doctor was not physically next to us to correct us”*. 16/56(28.5%) students in the DL group explicitly expressed their preference of the F-F approach for learning practical skills: “*I prefer to have had it face-to-face since I’d get into concept faster”*, *“I would have preferred if it was face-to-face”.*

## Discussion and literature review

### Terms that indicate the use of technology in education

Different authors have used various terms to indicate the integration of technology into educational activities; however, these terms are sometimes conflicting, serve certain branding agendas, or “lack conceptual clarity” ([26]; p 132). Popular terms include electronic learning (e-learning), online learning, digital education, remote learning, distance learning, and distance education, with many of these terms often used interchangeably despite the intricate differences between them [26, 51]. E-learning is often used to indicate the utilization of electronic resources, such as the computer and internet, to facilitate meaningful learning [40] whereas distance learning appears to indicate the utilization of e-learning tools outside the confinements of the physical classroom [4]. Face-to-face, also known as traditional or classic, education implies that the content delivery happens through bidirectional and real-time physical and mental interaction between the tutor and the learners under the same ceiling. Fawns et al. [27] argue that digital tools, such as videos and mobile technologies, have long diffused into the ‘traditional’ classroom thus making the precise distinction between digital and non-digital education unrealistic and unnecessary.

In what follows, we have used the term “**e-learning**” to indicate the utilization of electronic resources as instructional tools regardless of the physical location of the learners (remotely or within the physical classroom), and “**distance learning**” to indicate remote delivery of the educational content without the physical co-presence of the tutor and learners in the same classroom. We used the term “**face-to-face**” to indicate the delivery of the educational content within the confinements of the physical classroom regardless of the technology used for instruction.

### Applications of distance learning and telesimulation in surgical education

Various e-learning tools, such as videos, are commonly utilized in face-to-face educational sessions as well as in remote settings, whereby learners can access the resources from any location with the potential advantages of equity, cost-effectiveness, and time-flexibility [42]. Distance learning is defined as “providing access to learning for those who are geographically distant” ([51]; p 129). Distance learning has been utilized to deliver the cognitive as well as the practical components of education in various fields including social and healthcare sciences [64] as well as in surgical education [60].

Three distinct generations of distance education have been identified namely non-interactive, interactive, and virtual learning environments [46]:

1. Non-interactive web-based videos, audio-clips, and digital texts are tools for distant self-learning, they are ideal for conveying knowledge component of education; however, they do not involve any direct input from a tutor.
2. Teaching practical skills is challenging and requires that a tutor directly observes learners and corrects their mistakes in an iterative manner until they reach the desired competency level [64]. This may be achieved remotely if a mechanism for feedback and monitoring is made possible such as via teleconferencing platforms [44] that allow bidirectional real-time communication between the learners and tutors, thus called interactive distance learning.
3. Virtual learning environments encompass the combination of synchronous and asynchronous interactions with peers and tutors in addition to various multimedia and digital resources [46].

The feasibility of the provision of asynchronous (delayed) feedback based on recorded videos of performances have been explored, although not extensively, in the context of surgical education. In their RCT, Al-Jundi et al. [3] concluded that e-feedback based on video-assessment of suturing skills is acceptable and considered beneficial by the students, and that the quality of the e-feedback does not significantly differ from that of the classic face-to-face feedback. The authors utilized validated OSATS to measure their outcomes; however, the study involved only 37 students, and the power calculation was omitted, hence the poor generalizability of this study’s results.

Telesimulation is a relatively new educational tool that combines the concepts of simulation and teleconferencing to teach practical skills; it allows the direct monitoring and real-time communication between the tutor and the learners in simulated geographically distant environments [48]. There have been recent growing interests in the application of distance learning in surgical education, both at the undergraduate and post-graduate levels [71] with multiple successful attempts at utilizing telesimulation in teaching surgical knowledge and skills to overseas learners in underdeveloped and underserved countries [7, 43, 56].

The COVID-19 pandemic and subsequent social distancing and lockdown mandates have brought distance education in various fields including surgical education to the spotlight [47, 49, 70] in an attempt to limit the spread of the virus, and protect the students, faculty members, and patients, as well as to preserve personal protective equipment. Few authors have described their experience in teaching basic surgical technical skills remotely during the COVID-19 era utilizing video-conferencing technology [13, 15, 34] with preliminarily encouraging outcomes; these papers however were non-experimental, did not include control groups, did not utilize standardized tools for assessment, and did not offer statistical analysis of the results. The acceptability and effectiveness of these methods in surgical education remain largely unknown.

### The participants

Most of the participants of this study belong to generation-Z as they were born between the late 1990s and early 2000s; this generation is known to be tech-savvy [50], which may explain their rapid adaptation to the new technology-based methods of instruction [2].; p 263) considered that the effectiveness of “technology-driven learning and teaching experiences … could be significantly affected by technological expertise”*.* Prior to their exposure to this study, and because of the COVID-19 pandemic, all the participants have had some experience with online learning; however, their experience was limited to the cognitive domain of learning, and this was their first experience with remote learning of surgical practical skills.

Personal factors such as playing musical instruments and hand-dominance were not significantly different between the two groups of our study and hence excluded as confounding factors. The notion that playing musical instruments makes learning technical surgical skills easier and more efficient has been recently brought to the attention of clinical educators with more surgical training programs giving credits to applicants with musical backgrounds during the recruitment process [33] due to presumed superior dexterity and hand-eye coordination. Sun et al. [67] conducted a prospective cross-sectional trial on 51 novice students and showed that having a musical background is associated with significantly higher performance in learning basic surgical skills. The authors used validated tools for outcomes measurement and blinded the assessors to the participants’ groups thus minimizing observer bias. However, the voluntary participation in the study may have resulted in self-selection of the participants who are interested in learning surgical skills thus causing selection bias. Conversely, there have been few studies that highlighted potential difficulties in learning and assessing surgical skills in left-handed individuals [41].

### The teaching sessions

Despite the research team’s efforts to recruit 7–8 students for each session, the actual number of students per session ranged between 2 and 8, this is because of the COVID-19-related lockdown coupled with frequent road closures in the country due to political unrests during the study period. The number of students per session could have affected the efficiency of the sessions, however, the range and mean number of students per session was similar in the two arms of the study.

Face-to-face sessions were slightly more time-efficient (80.93 versus 89.39 s) despite not reaching statistical significance (*p* = 0.17); this could be related to the greater difficultly in explaining some of the steps that need visuospatial abilities such as proper technique of mounting the needle on the needle holder across the computer-screen. This could also be partially explained by the time spent on the technical adjustments during the DL sessions such as troubleshooting connection cut-offs and adjusting the camera angles. Another factor that may have contributed to this finding is that both the students and the tutor have more experience in face-to-face teaching of practical skills as compared to distance learning.

### The assessment tools

In general, few tools have been validated for the assessment of suturing skills in students [52], most of which incorporate similar themes such as proper use of the instruments, piercing the tissue at 90 degrees, crossing the knot, and time needed to complete a square knot [62]. We utilized two validated assessment tools to measure the students’ performances: A performance checklist by Sundhagen et al. [68] and an OSATS global rating tool [1]. Two independent assessors scored the performances of all the participants utilizing both assessment tools. We were able to demonstrate a satisfactory agreement between the two assessors for the two assessment tools by comparing the percentages of students who successfully demonstrated each of the individual checklist and OSATS sheet items, as well as the average checklist formula scores and OSATS total scores provided by the two assessors. Similarly, Sundhagen et al. [68] and Martin et al. [45] have reported acceptable inter-rater reliability for the checklist and the OSATS scores respectively that we used in this study.

### Objective performance outcomes

None of the participants across the two groups failed the assessment task; they all were successful in placing three secure interrupted sutures using sound basic surgical techniques. Overall, there were no statistically significant differences in the performance of the students between the two groups both at the level of the individual checklist items (*p*-values ranged between 0.27 and 1), the checklist formula scores (326.10 ± 42.54 versus 306.05 ± 65.03; *p* = 0.06), and the OSATS total scores (27.54 ± 5.99 versus 27.98 ± 5.24; *p* = 0.63). Hence, despite the fact that the participants in the F-F group slightly outperformed their peers in the DL group at the level of the checklist formula score, F-F and DL approaches seem to have comparable effectiveness in facilitating the learning of basic surgical skills in our cohort. This holds true even for the steps that were perceived as difficult to grasp remotely by the participants (and the tutor), such as mounting the needle on the needle holder. 48(81.4%) of the participants in the F-F group were able to demonstrate proper techniques of needle mounting versus 43(72.9%) in the DL group; the difference was not statistically significant (*p* = 0.27).

Our findings reflect the scarce published literature on the topic. The majority of the authors reporting on the difference in the students’ performance scores between face-to-face and remote teaching of basic surgical skills demonstrated no statistically significant differences between the two approaches [2, 53, 59, 69, 73].

The students who were randomized to the DL group were able to complete the assessment task faster than those randomized to the F-F group (282 s versus 248 s); the difference was statistically significant (U = 5596, *p* = 0.01), however, a difference of 34 s between the two groups is of little practical value.

### Participants’ perspectives

While the assessment of the participants’ performances by the two surgeons provided objective and measurable data, the questionnaires provided valuable subjective feedback on the process. Even though students’ scores were very similar in the two arms of the study, the subjective feedback hint towards a more tedious process in the distance learning group.

As detailed in the results section above, the distance-learning participants were generally highly satisfied and confident about their learned skills as their peers in the F-F group (Figs. 5, 6 and 7). This is congruous with the published literature with the majority of authors reporting on the acceptability and the positive students’ overall ratings of the distance learning of practical skills [2, 11, 17–19]. However, around half of the participants of our study in the DL group provided negative comments mostly related to the visuospatial challenges of learning surgical skills at a distance. Learning manual skills through a screen deprives students of the ability to look closer and at different angles at the working field. It more importantly transforms an essentially three-dimensional experience into a two-dimensional one. This makes it harder for the students to learn critical skills such as needle mounting and wrist motion. Other negative comments were related to the internet connectivity, despite being an institutional connection provided by the hospital. Okrainec et al. [57] reported on the issue of poor internet connectivity complicating distance learning particularly in the developing countries.

Around quarter of the participants in the DL group explicitly expressed their preference of the F-F approach when it comes to learning practical skills [30]. reported similar concerns related to the shortcomings of remote learning of surgical skills and documented their participants’ preference for the traditional face-to-face approach. Corrêa et al. [21] also reported similar results; most of their participants positively rated the distance learning sessions despite the fact that 50% of them preferred F-F instruction for learning practical skills. On the other hand, Bello et al. [9] lie on the opposite side of the spectrum as they reported higher satisfaction rate in the DL arm of their trial.

The learning experience is a two-way process between the tutors and students. Hence, the feedback of the instructor is a valuable addition to the evaluation of the two approaches in question. Although we did not include an official evaluation of the sessions by the tutor, my teaching experience closely mirrored those expressed by the students in the comments section. Even though the end-result in terms of the participants acquiring the skills was very similar between the two branches of the study, the effort to help them reach the required competencies was greater in the distance group. Certain skills such as needle mounting and taking the correct bite depth were particularly hard to convey across the screen.

### Is distance learning feasible, acceptable, and effective in acquiring practical surgical skills?

In their questionnaire-based observational study that included 22 participants, Corrêa et al. [21] concluded that distance learning of practical skills is feasible, useful, and acceptable by the undergraduate dental students, however, 50% of the participants expressed their preference of getting immediate face-to-face feedback while learning basic dental surgical procedures. In addition to the small sample size and biases intrinsic to the survey-based studies, the authors utilized a non-validated questionnaire for outcome measurement.

In their randomized case-control trial, Kumagai et al. [39] showed that distance learning is feasible and effective in acquiring endoscopic sinus surgical skills in novices; the study, however, did not include a control group, had a small sample size (17 participants), and the authors did not utilize a validated tool for outcome measurement. Mosalanejad et al. [53] showed, in a prospective nonrandomized trial involving 86 participants, that remote learning of practical skills in novice nursing students using video technology and virtual animations is effective and comparable to the traditional face-to-face methods, with superior knowledge acquisition outcomes in the distance learning group. The authors used a validated checklist to measure their outcomes, however, they did not explicitly describe the nature of the practical skills they studied, and they did not include a baseline assessment of the students’ competency at the commencement of the trial. Van Duijn et al. [69] included 53 physical therapy students in a prospective randomized crossover trial to compare face-to-face instruction to web-based videos in teaching practical skills. The authors found no statistically significant differences between the two groups; however, they observed significant improvement in the outcomes after the students’ crossed groups, which indicates beneficial effect of exposure to both methods of instructions (hybrid methods). The authors used non-validated tools for outcomes measurement and did not monitor the participants’ practice time outside the study; this might have opened doors for bias [65].

In their prospective randomized trial, Ali et al. [2] showed no differences in the outcomes between face-to-face and telesimulation groups in learning advanced trauma life support (ATLS). The authors utilized validated ATLS practical skills evaluation sheets for outcomes measurement, however, their sample size was relatively small (30 participants), and the assessors were not blinded to the participants’ group which may have opened doors for observer bias [31]. Similarly, Winder et al. [73] showed, in a prospective randomized trial including 34 veterinary students, no differences between face-to-face and distance learning of cornual nerve block and disbudding of dairy calves in terms of successful completion of the tasks; however, the distance learning group showed superior knowledge acquisition but less confidence and poorer technical skills as compared to the face-to-face group. The authors utilized a validated checklist for practical skills assessment; however, they used a non-validated written test for knowledge assessment and a non-validated questionnaire for the evaluation of the participants’ confidence. Another prospective randomized trial [7] showed that distance learning of double-handed knot tying in novice surgical trainees is feasible, effective, and acceptable both by teachers and by learners; the authors used a validated OSATS to measure their outcomes, however, the sample size of 18 was small.

Most of the published papers on the topic conclude that distance learning is feasible and effective in the context of learning practical skills in novices, with some authors providing evidence of superiority of distance learning in acquiring surgical knowledge over the traditional approach [53, 73]. There is also a near-consensus on the acceptability of distance learning with the majority of the participants across the studies positively rating this approach [2, 9, 11, 18], despite few papers presenting evidence of learners’ preference of the face-to-face instruction for learning practical skills [21, 30].

No clear guidance exists in the literature on how to best approach remote teaching of surgical technical skills [22]. Most of the published literature explore the acceptability and effectiveness of the computer and web-based instructional methods in surgical education [6, 10, 55]; most of these studies show that e-learning tools constitute a useful adjunct in surgical education that cannot, and is not meant to, replace the traditional methods and approaches [24]. Several authors tried to evaluate the feasibility of distance learning in the non-technical aspects of surgical education [43]; the results were mostly encouraging. Only few trials explored the effectiveness of distance learning in the acquisition of psychomotor surgical skills in novice learners, almost all these studies concluded that distance learning is feasible, acceptable, and effective in this context. However, many of these papers have intrinsic methodological weaknesses such as the reliance on non-validated tools for outcomes measurement and small sample sizes. A recently published systemic review [17, 19] has confirmed the feasibility of the remote approach in learning surgical skills in novices, with no difference as compared to the classic approaches in some trials, and reduced task-completion time and increased accuracy in the student performance in other studies.

To our knowledge, there are no published adequately powered prospective randomized trials that compare the acceptability and effectiveness of face-to-face learning to distance learning of basic suturing skills in novices, assessed by two blinded assessors utilizing validated tools for outcomes measurement.

### How different is the DL from the F-F approach?

Distance learning in health education is an evolving field. Most of the published literature draw an optimistic outlook; it is thought to be effective, efficient, cost effective, and convenient for the learners as compared to the traditional methods of instruction [46, 72]. That said, most of these studies are survey-based and focus on the learners’ perceptions and attitudes [46] rather than on objective outcomes measurement. On the other hand, [23]; p 11) considers that “distance learning has failed” because it disconnects the learners from their physical and emotional environments which are indispensable for learning. This perspective was challenged by [27]; p 293), who view online learning as “embodied, socially meaningful experience” and advocate for blurring the sharp distinction between online and face-to-face learning. The authors argue that the two approaches, though intrinsically different, often intersect in many aspects, and the actual learning process continues beyond the physical confinements of the teaching sessions regardless of the instructional approach.

Post digital education is a contemporary philosophical stance towards online learning that blurs the distinction between electronic (digital) learning, and face-to-face learning [26]. Proponents of the postdigital perspective believe that all kinds of learning (digital or not) utilize both physical interactions and electronic tools and, if meaningful and relevant, are all socially and emotionally embodied regardless of whether the educational events happen under the same roof or not [26]. Considering this research project for instance, we have utilized video illustrations and live demonstrations of the suturing skills in both arms of the study, face-to-face in one group and through videoconferencing in the other. The students were able to interact and receive immediate feedback from the tutor regardless of whether he was physically present in the same room or not. The open/flexible and mastery learning pedagogic principles and modelling and explaining instructional strategy outlined above apply to both groups of the study; in addition, student-centeredness, explicit illustration, and deliberate practice of the suturing skills were exercised in both groups.

Distance learning is not a mere process of uploading face-to-face educational content on the internet [27]; each method requires specific set of expertise and skills, with [27]; p 139) arguing “they (online courses) must be (re) designed to take cultural and technological contexts into account”. A lot of effort went into designing and delivering the online sessions of this study to adapt to the specific challenges of the teleconferencing technology. There were several challenges specific to the remote group, such as accurate camera positioning to allow optimal visualization of the students’ hands while suturing, maintaining active engagement of the participants in the absence of direct eye contact, and explaining the principles of mounting the needle on the needle holder that require visuospatial cognitive and technical processing on the students’ side. The needle mounting process was particularly easier to teach face-to-face because the tutor was able to physically help the students with the mounting process in the initial phase of learning, which was not feasible in the distance group. That said, and despite being more challenging, telesimulation was as effective and efficient as the face-to-face method in teaching basic suturing including the difficult steps such as mounting the needle on the needle holder and was generally reviewed favourably by the participants despite their sceptical comments regarding the difficulty of grasping some of the steps that require visuospatial capabilities.

After all, distance learning is not expected to totally replace the hands-on experience in learning basic surgical skills, it can be utilized as an adjunct in this setting. Several potential benefits of direct face-to-face interactions with an expert tutor may not be amenable to measurement such as role modelling and acquiring technical nuances [9].

### Strengths and weaknesses of the study

This is a prospective and randomized trial; thus, information and selection biases were kept to a minimum [65], whereby data collection did not rely on the participants’ recalling abilities or previously recorded incomplete information, and the participants’ group-assignments were randomly chosen thus preventing skewing the results based on the participants’ personal characteristics. Assessors were blinded to the participants’ study groups thus avoiding observer bias [31]. Valid and reliable tools were chosen for outcomes measurement (participants’ performances) to prevent systematic measurement errors [20]. All the participants in both groups did not have any previous experience in basic suturing and were exposed to the same instructional video at the beginning of the sessions, which were all facilitated by the same instructor thus minimizing study confounders. The target number of participants was reached hence providing adequate power to the analysis.

Despite of the above strengths, we are aware of the several limitations of this study; this is a single-centre trial whereby all the participants were recruited from the same university hospital; hence our results may not be representative of the wider community of pre-medical, first and second-year medical students. We did not measure the participants’ baseline knowledge and skills levels; however, we recruited only the students who self-reported, via the questionnaire, no previous exposure to basic suturing. Even though we did not provide the participants with the illustrating videos before the scheduled sessions, some participants may have accessed and watched the same or similar videos available on the web; we did not monitor or control for this variable. Because of scheduling difficulties related to the lockdown during the data collection phase of the study, the number of students per session, in both groups, varied between two and eight students. This may have acted as a confounding factor, whereby the group size per session could have affected the efficacy of the learning process. We opted to conduct the distance-learning sessions within the hospital premises so that we use the reliable institutional Wi-Fi to avoid the expected sketchy private internet connectivity of some participants as a confounding factor, however, this may not be an accurate representation of real-life telesimulation, where students can join the teaching sessions from the comfort of their own homes. We did not account for the mirror image that the learners received, via videoconferencing, during the demonstration of the skills by the tutors; this may have affected the ability of the students to learn the skill. We have utilized a non-validated questionnaire to evaluate the participants’ confidence and satisfaction. Additionally, we used the learners’ satisfaction and their competency levels in a simulated setting to compare the effectiveness of face-to-face versus distance learning of basic suturing; this matches level two on the Kirkpatrick’s hierarchy [36, 74]. Our study, however, does not provide an insight to the impact of the delivery method pertaining to the higher levels on the Kirkpatrick’s hierarchy, such as translation into actual performance in real-life situations and impact on patients’ outcomes. Finally, we did not attempt to evaluate the difference in the retention rate of the acquired skills over time between the two groups; this is due to the delays in data collection caused by the extended lockdown periods in the country.

## Conclusion

Based on our findings, distance learning of simple interrupted suturing is as effective and efficient as the traditional face-to-face approach in novice pre-medical and first and second-year medical students. It is acceptable and perceived as beneficial and enjoyable by the students despite having expressed their preference of the face-to-face approach due to the difficulties and challenges related to remotely learning technical visuospatial concepts.

Distance learning can be used as an adjunct to the traditional face-to-face approach in teaching basic surgical skills to novices. It can also be utilized as an effective alternative to the traditional instructional methods, for instance during pandemics or for teaching practical surgical skills to individuals in remote understaffed locations thus potentially helping in improving the quality of healthcare in the developing parts of the world.

## Data Availability

The datasets generated and/or analysed during the current study are not publicly available due to the Institutional Review Board requirements but are available from the corresponding author upon a reasonable request.
